# Chest pain prevalence, causes, and disposition in the emergency department of a regional hospital in Pretoria

**DOI:** 10.4102/phcfm.v8i1.1048

**Published:** 2016-06-10

**Authors:** Mimi Geyser, Selma Smith

**Affiliations:** 1 Department of Family Medicine, University of Pretoria, Pretoria, South Africa

## Abstract

**Background:**

Chest pain is a common clinical syndrome. However, there is a paucity of African studies describing the causes, prevalence, aetiology, and disposition of patients with chest pain presenting in the emergency department (ED).

**Aim:**

The aim of this retrospective descriptive study was to determine the prevalence, causes, demographics, and disposition of all adult patients with the main complaint of chest pain presenting at the ED of a regional hospital in South Africa.

**Methods:**

Records of all patients 18 years and older presenting with the complaint of chest pain from 1 December 2011 through 10 April 2012 were assessed. A data collection sheet capturing patient demographics and disposition from the ED was used. The diagnosis was subdivided into groups: cardiovascular, respiratory, gastrointestinal, musculoskeletal, psychiatric/psychogenic, other, and unknown.

**Results:**

Of the 312 patients presenting with chest pain, 210 patient files were retrieved. The prevalence of non-traumatic chest pain was 1.66%. Respiratory disease was the most common cause (36.19%), with pneumonia the most common diagnosis (24.40%). Logistic regression showed diagnoses of acute cardiovascular disease or respiratory disease, older age, and transport by ambulance as being associated with admission.

**Conclusion:**

The main cause of acute chest pain was found to be respiratory disease, followed by musculoskeletal disorders. In the African context, the aetiology of acute chest pain differs from that in first world countries. Health workers should therefore pay special attention to respiratory conditions during diagnosis and management in African patients with acute chest pain.

## Introduction

Chest pain is quite common and ‘up to 25% of the general population experience it’ in some form during their lifetime.^1,2^ In a Belgian study,^3^ the prevalence of chest pain in the emergency department (ED) was reported to be 2–5%. As expected, the prevalence in primary care is less, with an estimation of 1–2%.^4,5^

Acute chest pain is a clinical syndrome that may be caused by almost any condition affecting the thorax, abdomen, or internal organs.^4^ It may be broadly categorised as somatic or visceral in origin. Somatic pain includes pain from the musculoskeletal structures, coverings of major organs, and dermal tissues, whereas visceral pain includes pain from structures like the heart, stomach, and so forth.^5^

When a patient presents with chest pain in the ED, the first priority is to consider potentially life-threatening causes.^6^ These causes are acute coronary syndromes, aortic dissection, pulmonary embolism (PE), ruptured aortic aneurysm, and tension pneumothorax.^7^ The most common of these is myocardial infarction (MI), or ischaemia.^8^ In patients with non-life-threatening chest pain, it may be only after a complete workup, including a comprehensive history, physical examination, and some further investigations, that a diagnosis is reached.^7^

Cardiovascular conditions (MI, angina, PE, heart failure) are diagnosed in more than half of patients presenting to the ED with chest pain.^3^ Then again, 50–80% of patients are discharged without a diagnosis or with a diagnosis of a non-cardiac condition.^3,5,8^ Non-cardiac chest pain is defined as chest pain that is not related to coronary artery disease. Population-based studies on the epidemiology of non-cardiac chest pain have reported that the prevalence of non-cardiac chest pain in the community ranges from 23 to 33%,^1,9^ and there seem to be no differences between males and females. Furthermore, roughly 2–5% of all presentations to hospital EDs are for non-cardiac chest pain.^1^

The diagnostic approach to chest pain, which is ‘a “risk avoidance” and a “rule out coronary heart disease” strategy,^3^ might improve with better knowledge of the wide spectrum of etiology of non-cardiac chest pain’.^5^ These conditions include musculoskeletal syndromes, pulmonary disease, psychological disorders (panic attacks, anxiety, or somatisation) and disorders of abdominal viscera.^6,8^

A confident diagnosis of musculoskeletal chest pain can be challenging because no clear reference standard exists.^5^ In primary care, musculoskeletal conditions account for 21–49%^5,10^ of causes of chest pain, but only 7% in the ED.^10^

Pulmonary diseases account for 12–14%^3,10^ of the patients with chest pain in the ED, with PE, pneumonia, spontaneous pneumothorax, and pleuritis as main causes.^8^

Studies have reported that patients with non-cardiac chest pain and normal coronary arteries suffer from various psychiatric disorders, most commonly panic disorder, anxiety, and depression.^1,8^

An audit showed that overall 61% of patients with chest pain of all causes were assessed and discharged home by ED doctors without recourse to a second opinion. Of patients thought by the ED doctors to have chest pain of cardiac origin, who were referred to the duty medical registrar or cardiologist, 88% were admitted.^2^

The literature usually focuses on how to evaluate patients with acute chest pain in different clinical settings, but few articles describe the range of conditions that present with chest pain. In addition, there is a lack of South African studies on the prevalence, aetiology, and disposition of patients with chest pain presenting in the ED.

## Aims

The aim of the current study was to determine the prevalence, causes (defined as clinical diagnosis at disposition from the ED), demographics, and disposition of all adult patients with the main complaint of chest pain presenting at the ED of a regional hospital in South Africa.

## Methods

A retrospective descriptive study of all ED patients with chest pain was done at the ED at Kalafong Hospital, to which 49 200 new patients present every year. Kalafong Hospital is an academic hospital affiliated to the University of Pretoria. It delivers mostly secondary-level services with some primary and tertiary services available. Most of the population that Kalafong serves have no access to a community health centre or district hospital, and so Kalafong is the only after-hours medical service available to them.

The study population consisted of all patients 18 years and older with the main complaint of chest pain from 1 December 2011 through 10 April 2012.

Records of all patients presenting with the complaint of chest pain in the time frame were assessed. A data collection sheet was used. Data included patient demographics and disposition from the ED. The diagnoses were subdivided into the following groups: cardiovascular, respiratory, gastrointestinal, musculoskeletal, psychiatric/psychogenic, other, and unknown. Cardiovascular causes were subdivided into ischaemic and non-ischaemic diseases. Somatoform disorders constituted a group of psychiatric disorders with predominantly physical symptoms that were not fully explained by a general medical condition.

### Data analysis

Univariate statistical analysis was done to assess the characteristics and demographics of participants. Stata software (Intercooled Stata 8.1 for Windows; StataCorp, College Station, TX, USA; 2003) was used for data analysis. Student’s *t-*test was used for continuous data. For multivariate analysis, logistic regression was used. All variables identified in the univariate analysis with *p*-values up to 0.2 were tested in the model to determine significance and identify confounders.

### Ethical considerations

The ethics committee of the Faculty of Human Health Sciences of the University of Pretoria approved the study (approval number: 174/2011). The study was carried out according to the ethical principles of the Declaration of Helsinki and the Declaration of Geneva of the World Medical Association.

## Results

The prevalence of chest pain in the ED was found to be 1.82% (traumatic and non-traumatic). The prevalence of non-traumatic chest pain was 1.66%. According to the ED register, 312 patients with chest pain presented between 1 December 2011 and 10 April 2012. The characteristics of these patients are presented in Table 1.

**TABLE 1:** Characteristics of study participants.

Characteristics	% (*n*)
**Gender**	
Male patients	53.70 (167)
Female patients	46.30 (144)
Ethnicity	-
African people	75.24 (234)
White people	18.33 (57)
Indian people	3.54 (11)
Other	2.89 (9)
**Transport**	
Own/public	61.93 (109)
Ambulance	36.93 (65)
**Disposition**	
Admitted by specialists	33.33 (104)
Transferred to Steve Biko	0.32 (1)
Academic Hospital	56.09 (175)
Discharged by ED doctor	7.37 (23)
Discharged by specialist	0.96 (3)
Died in ED	-
Absconded/refused treatment	1.92 (6)

*Source*: Authors own work

ED, emergency department; Age (y), *N* = 312; Mean ± s.d., 42.41 ± 16.48.

Of the 312 patients, it was possible to retrieve 210 patient files. Respiratory disease (Table 2) was the most common cause of chest pain (36.19%) and pneumonia was the most common diagnosis (24.40%). Musculoskeletal problems (21.90%) and cardiovascular disease (21.43%) were the other most important causes. Within the cardiovascular disease category, ischaemic heart disease (13.81%) was the most prominent component. Trauma was the cause in 9% of chest pain patients.

**TABLE 2:** Causes of chest pain and disposition.

Cause	% (*n*)	Admitted% (*n*)	Discharged% (*n*)	OR 95% CI	*p*-value
**Cardiovascular**	21.43 (45)	43.16 (41)	3.48 (4)	21.07 [7.01–83.91]	< 0.0001
Ischaemic heart diseases	13.81 (29)	-	-		-
Angina	2.39 (5)	-	-	-	-
Unstable angina	1.91 (4)	-	-	-	-
STEMI	3.35 (7)	-	-	-	-
NSTEMI	6.22 (13)	-	-	-	-
Heart failure	3.93 (8)	-	-	-	-
Arrhythmia	0.48 (1)	-	-	-	-
Acute pericarditis	1.44 (3)	-	-	-	-
Other	1.91 (4)	-	-	-	-
**Respiratory**	36.19 (76)	46.32 (44)	27.83 (32)	1.91 [1.03–3.57]	0.0275
Pneumonia	24.40 (51)	-	-		-
PE	0.96 (2)	-	-	-	-
Pneumothorax	0.48 (1)	-	-	-	-
Pleurisy	2.87 (6)	-	-	-	-
Pleural effusion	1.91 (4)	-	-	-	-
Other	5.74 (12)	-	-	-	-
**Gastrointestinal**	6.67 (14)	1.05 (1)	11.30 (13)	0.08 [0–0.58]	0.003
**Musculoskeletal**	21.90 (46)	3.16 (3)	37.39 (43)	0.05 [0.01–0.18]	< 0.0001
Costochondritis	11.43 (24)	-	-		-
Trauma	9.09 (19)	-	-	-	-
Other	1.44 (3)	-	-	-	-
**Psychological/psychiatric**	3.33 (7)	-	6.09 (7)	0 [0–0.63]	0.01
**Unknown**	6.67 (14)	1.05 (1)	11.30 (13)	0.08 [0.00–0.58]	0.003
	-	-	-		-
**Other**	3.81 (8)	5.26 (5)	2.61 (3)	-	-

*Source*: Authors own work

OR, odds ratio; CI, confidence interval; STEMI, ST-elevation myocardial infarction; NSTEMI, non-ST-elevation myocardial infarction; PE, pulmonary embolism.

Other causes of chest pain were diabetic ketoacidosis, urinary tract infection, alcoholic liver disease, renal failure, pyelonephritis, and intracranial bleeding. Respiratory disease (46.32%) and cardiovascular disease (43.16%) were also the most common cause for admission by specialists. Of the patients admitted with respiratory disease, 70.45% (31/44) were diagnosed with pneumonia. Of the pneumonia patients, 29.4% were known to be HIV positive before admission. Pulmonary tuberculosis was diagnosed in 7.8% of patients with respiratory disease.

Regarding age, patients with cardiovascular disease tend to be older than the average age, with a mean age of 55.07 (SD 16.04) years. Patients with psychological disorders were younger than average, with a mean age of 29.86 (SD 6.79) years. The mean age for patients with respiratory disease was 43.21 (SD 16.58) years and for musculoskeletal problems 35.74 (SD 10.83) years.

Cardiovascular disease patients were more often male (66.67%) and utilised ambulance transport (53.66%) more frequently than patients with other causes of chest pain.

Patients with psychological disorders were most often females patients (85.71%) and were transported to hospital by their own or public transport (80%). Co-morbidities of hypertension (61.36%) and diabetes mellitus (13.63%) were the most prominent in the cardiovascular disease group.

**FIGURE 1:**
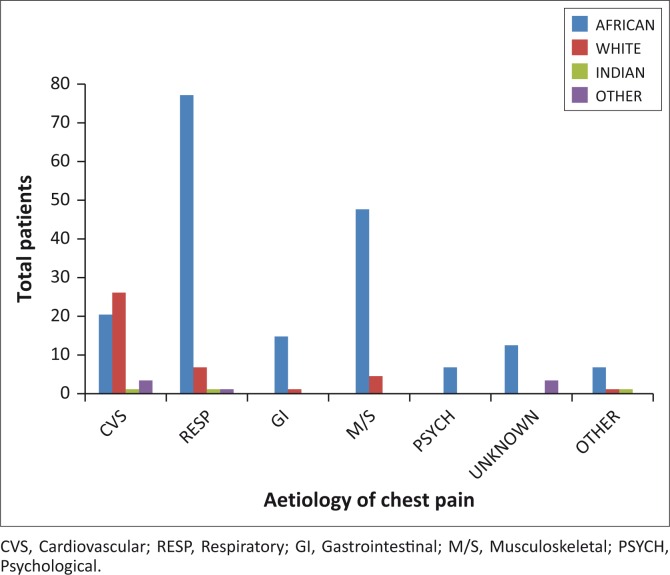
Aetiology of chest pain vs ethnicity in ED patients.

African patients were dominant in all the aetiology groups except cardiovascular disease (40%), where white patients were more prevalent (51%) (Figure 1).

Regarding disposition, 63.46% of patients were discharged, of which 56.09% were discharged by the ED doctors. A third of patients were admitted to the hospital, one patient was transferred to a central hospital, six patients absconded, and three died in the ED. The odds of being admitted were highest for patients with cardiovascular disease (odds ratio (OR) 21.07, 95% confidence interval (CI) [7.01–83.91]; *p* < 0.0001), with a probability of 91%. Patients with respiratory disease were more likely to be admitted than discharged (OR 1.91, 95% CI [1.03–3.57]; *p* = 0.03), with a probability of 58%. The odds of being discharged were the highest for musculoskeletal disorders (OR 0.05, 95% CI [0.01–0.18]; *p* < 0.0001), with a probability of 93.48%.

Further analysis by logistic regression was done to assess clinically relevant and statistically significant variables identified in the univariate analysis regarding disposition. The final model (Table 3) consisted of the dependant variable (disposition), diagnosis of acute cardiovascular disease, diagnosis of respiratory disease, diagnosis of musculoskeletal disease, age, and mode of transport with the odds ratio 26.86; *p* < 0.0001. Forward and backward analyses were conducted. There was no interaction between the variables.

**TABLE 3:** Factors associated with disposition of chest pain patients (multivariable model).

Factor	*p*-value
Diagnosis of cardiovascular disease	< 0.0001
Diagnosis of musculoskeletal disease	< 0.0001
Age	0.002
Diagnosis of respiratory disease	0.02
Mode of transport	0.03

*Source*: Authors own work

## Discussion

This was a retrospective descriptive study, investigating the overall case mix of patients presenting with traumatic and non-traumatic acute chest pain in an academic hospital ED in South Africa. The aim of our study was to describe the prevalence, spectrum of causes, demographics, and disposition of acute chest pain patients specifically in an African context. To date, this was the first African study on the aetiology of acute chest pain with major differences to first world publications.

The prevalence of chest pain in the unit was 1.66% for non-traumatic chest pain - lower than the 2.7% found in a Belgian study.^3^ The mean age of chest pain patients was 42.41 years, much younger than in other studies, for example Knockaert et al.^3^ and Buntinx et al.^10^ with a mean age of 60 years, Henderson et al.^11^ with a mean age of 53 years, and Aguilera et al.^12^ with a mean age of 53 years. This difference could be explained by the high prevalence of respiratory disease (mean age 43.21 years [SD 16.58]) and musculoskeletal disorders (mean age 35.74 year [SD 10.83]) found in our patients. The unique profile of our chest pain patients may partly be a result of our setting, as there is no district hospital to serve patients with primary-level respiratory and musculoskeletal disorders needing hospitalisation or after-hours care. Our subgroup of patients with cardiovascular disease had a mean age of 55 years, however, which is comparable to the other studies’ findings.

The most common cause of acute chest pain was found to be respiratory disease (36.19%), followed by musculoskeletal conditions (21.9%) and cardiovascular disease (21.43%). Our finding regarding the prevalence of respiratory disease (36.19%) was in contrast to all other studies: Knockaert et al.^3^ found 14.2% and Buntinx et al.^10^ 12.1%, respectively. The high occurrence of respiratory disease in patients with chest pain in our unit may be explained by the high prevalence of HIV. Regarding demographics, 41.5% of our black African patients had respiratory disease. In comparison, Aguilera et al.^12^ also found cardiovascular disease at 20.5%, in contrast with Lee and Goldman^13^ (45–50%), Knockaert et al.^3^ (51.7%), Buntinx et al.^10^ (54.3%), and Fothergill et al.^2^ (41%).

Most of our patients were black Africans (75%), but most of the patients with cardiovascular disease were white people (51%). The proportion of musculoskeletal conditions (21.9%) found in our study compared well with that of Ayloo et al.,^14^ who found a prevalence of 21–49%, but differed from other studies, which found 7%.^3,10,14^

The proportion of transport by ambulance (37%) concurred with other studies.^3^ In particular, cardiovascular patients were transported more by ambulance than by their own or private transport, similar to a study done in Gothenburg.^15^

Regarding disposition, 56% of chest pain patients were managed and discharged by the ED doctors without recourse to a second opinion. This figure falls within the widely differing findings of Henderson et al.,^11^ where only 15.6% of patients were discharged, and Fothergill et al.,^2^ who found 61% were discharged. Ultimately, 33% of all chest pain patients were admitted.

Logistic regression in our study identified diagnosis of acute cardiovascular disease, diagnosis of respiratory disease, older age, and transport by ambulance as being associated with admission to a hospital ward, whereas diagnosis of musculoskeletal disorders was associated (93.8%) with discharge from the hospital. Only 9% of patients with cardiovascular disease were discharged, in comparison to 42% with respiratory disease. Admitted patients mostly had respiratory disease (43%). These findings are very different from Aguilera et al.,^12^ where 76% of admitted patients had cardiovascular disease and 11% had respiratory disease.

Although 112 patient files could not be retrieved, data on a large number of variables were available from the emergency unit register, including disposition. The decision was made not to remove those cases with incomplete data, as it would produce biased parameter estimates. The sample of 210 complete patient records was sufficient to achieve statistical power of more than 80% for the study.

### Limitations

Diagnoses were often made based on clinical assessment with limited special investigations or specialist input. It is problematic that the diagnosis of gastrointestinal disease was made after clinical assessment and limited investigations, especially in discharged patients. Psychological diagnoses were also made without the opinion of a specialist in the field.

Although the power of the study was sufficient, the study period was relatively short and a year might have given more reliable results.

## Conclusion

The main cause of acute chest pain in the ED of Kalafong Hospital, South Africa, was found to be respiratory disease, followed by acute cardiovascular disease. The mean age of chest pain patients was younger than in any other study. The probability of being admitted was highest for patients with acute cardiovascular disease, acute respiratory disease, older age, and patients transported by ambulance, whereas the probability of being discharged was the highest for patients with musculoskeletal disorders.

In the African context, the aetiology of acute chest pain differs widely from first world countries. Health workers should therefore pay special attention to respiratory conditions during diagnosis and management in African patients with acute chest pain.
